# Perceived Mental and Physical Fatigue, Stress and Recovery, and Workload in Masters Athletes Pre‐ and Post‐Sailing Competition

**DOI:** 10.1002/ejsc.70082

**Published:** 2025-12-20

**Authors:** Sophie L. McIntosh, Lyndell M. Bruce, David P. Broadbent

**Affiliations:** ^1^ School of Exercise and Nutrition Sciences Deakin University Geelong Australia; ^2^ Centre for Sport Research Institute for Physical Activity and Nutrition Deakin University Geelong Australia

**Keywords:** competition, NASA‐TLX, perceptual fatigue, sport, SRSS

## Abstract

The aim of this study was to examine the effect of sailing competition on perceived mental fatigue and physical fatigue, stress and recovery, and workload for Masters athletes, and to explore the underlying modulating factors of perceptual fatigue. Using a cross‐sectional study design, 22 Masters athletes competing in the 2023 Oceania and Australian Laser Masters Championships completed a survey 48 h prior to and following the competition. The survey asked sailors to report on their perceived mental fatigue and physical fatigue (Visual Analogue Scales), stress and recovery (Short Recovery and Stress Scale), workload (NASA Task Load Index), and modulators of mental fatigue and physical fatigue. Wilcoxon Signed Ranks Tests were used to assess differences before and after the competition period. Mental fatigue and physical fatigue significantly increased due to the sailing competition, and this was accompanied by significant increases in stress and workload, and reduced recovery. Weather and complex decision making were identified as important modulators of perceptual fatigue during competition with weather having a larger influence on physical fatigue than mental fatigue. In conclusion, findings indicate a potential relationship between sailing competition and athletes perceived levels of mental and physical fatigue, and this is underpinned by changes in stress, recovery, and workload. Practitioners are encouraged to use self‐report measures to monitor these mechanisms and inform the development of individualised interventions that holistically manage perceptual outcomes.

## Highlights

- Mental fatigue and physical fatigue significantly increased due to the sailing competition in Masters athletes
- This was accompanied by significant increases in stress and workload, and reduced recovery
- Weather and complex decision making were identified as important modulators of perceptual fatigue during competition
- Practitioners are encouraged to use self‐report measures to monitor these mechanisms and inform the development of individualised interventions that holistically manage perceptual outcomes

## 1 Introduction

Sailing is a worldwide sport with a diverse participation profile, ranging from recreational to elite, and youth to Masters aged athletes. Performance requirements are specific to the class of boat and athletes' role (Bojsen‐Møller et al. 2007). The International Laser Class Association (ILCA) is considered one of the most popular Classes of boats in the world, sailed in over 120 countries. Technical and tactical competency, mental capability, and physical robustness are key performance parameters (Bojsen‐Møller et al. 2015; Brandt et al. 2016; Schultz et al. 2016). A typical competition consists of up to 12 races over 5–7 days, with each race lasting approximately 30–60 min (Sekulic et al. 2006; Tan et al. 2016). The format of scheduled races within this period remains flexible due to the unpredictability of weather conditions. The structure of individual racecourses also presents considerable physical and psychological stresses. Although course length and durations aim to remain within a specified range, implementation is dependent on prevailing weather conditions (Masino et al. 2021). Often, races are shortened or modified to align with the strength and direction of wind. The culmination of several races across multiple days, unpredictable weather conditions, and uncertainty in physical and cognitive demands of competition has a unique impact on perceptual fatigue, stress and recovery, and workload (Brandt et al. 2016; Sekulic et al. 2006; Tan et al. 2016). However, research in sailing is limited compared to high‐profile racing, team‐games or racquet sports. Expanding the current scope of literature will enhance coaching practices, sport science interventions, and subsequent athlete wellbeing and performance.

Perceptual fatigue is a construct of interactive behavioural processes requiring constant adjustment between the individual and environmental setting (Arruza et al. 2009). In a sports setting, this is evident through the imbalance of stress, recovery and/or workload resulting in reduced capacity to produce maximal performance (Kellmann et al. 2018). The role of measuring workload, stress and recovery in training and competition is key to understanding perceptual fatigue. Non‐invasive self‐report questionnaires are convenient, cost‐effective tools to assess perceived levels of fatigue (Nässi et al. 2017). Winchcombe et al. (2021) utilised heart rate (HR), global positioning system (GPS) and Short Recovery Stress Scale (SRSS) measures to quantify workload demands, internal stress response and perceptual fatigue experienced by elite ILCA sailors across multiple days of competition. HR and GPS were monitored during racing and the SRSS was completed at the end of each sailing day. The findings suggested that objective workload demands, and corresponding perceptual fatigue responses increased over time in more challenging regatta schedules. Whilst useful in building an understanding of workload and perceptual fatigue in sailing competition, this study examined perceptual fatigue as a single state, limiting the insight into the differing contribution of mental fatigue and physical fatigue parameters.

Contemporary fatigue frameworks, such as the State Fatigue Framework (Behrens et al. 2023), distinguish between mental (or cognitive) and physical (or motor) components of fatigue, as well as the corresponding subjective experiences of each parameter (i.e., perceptual fatigue). Research in sports such as netball (Russell et al. 2020), soccer (Smith et al. 2016) and rugby (Mariano et al. 2023) have supported this notion by demonstrating differences between the two aspects of fatigue and highlighting the importance of monitoring and managing them separately. This distinction is particularly relevant in sports like sailing, where athletes must sustain physical exertion (e.g., trimming sails, hiking) while simultaneously engaging in complex cognitive tasks (e.g., navigation, tactical decision‐making) during extended competition. Measuring these components separately allows for a more nuanced understanding of the phenomenon of fatigue and closer examination of how different fatigue states manifest, interact and impact performance from an objective and subjective perspective (Tornero‐Aguilera et al. 2022). Research interested in perceptual fatigue has utilised Visual Analogue Scales (VAS) to obtain data on athletes perceived mental fatigue and physical fatigue (Mariano et al. 2023; Russell et al. 2020). For example, Russell et al. (2020) distributed the VAS scale to 12 female netballers competing at the Australian Netball League. Perceived levels of mental fatigue and physical fatigue were recorded pre‐ and post‐match and were found to increase following competition. The authors suggested such findings could support player monitoring and development of strategies which manage the effect of fatigue on performance. Currently, the vast majority of previous research in sailing has employed single measures of perceptual fatigue and so generalises mental fatigue and physical fatigue parameters into the same entity (Winchcombe et al. 2021). Research is required to address these limitations and have mental fatigue and physical fatigue as the primary focal point.

Although the volume of research in sailing which examines mental fatigue and physical fatigue is increasing, the majority of studies utilise objective measures in simulated settings (e.g., Blackburn 1994; Bourgois et al. 2016; Caraballo et al. 2019). These studies may not accurately replicate the demands experienced in competition or consider the important role of perceptual fatigue. A mixed method approach that integrates quantitative measures of perceptual fatigue before and after actual competition with qualitative insights into athletes' personal experiences offers a novel opportunity to address existing gaps in the literature (Russell et al. 2024; Weiler et al. 2025). In the wider literature, there is also potential to extend understanding of the subjective experience by incorporating measures which consider individual expectations of upcoming events. More specifically, assessing the interaction of expected and actual demands and characteristics of competition (e.g., timings of different aspects of competition) would permit feedforward consideration of self‐regulatory processes influencing perceptual fatigue (Behrens et al. 2023). This would extend traditional assessments of perceptual fatigue which report perceived mental fatigue, physical fatigue, and workload responses without acknowledging the potential influence of athlete expectations (Behrens et al. 2023; Mariano et al. 2023; Russell et al. 2020).

Masters athletes represent a group of interest who have received limited attention in research, despite advances in participation and performance within the broader sport setting in recent decades (Lepers and Stapley 2016). Given Masters athletes will experience natural age‐related declines in physical capacity and cognitive function (Fell and Williams 2008; Reaburn 2021; Schmidt et al. 2021), it is important to investigate how fatigue manifests in this population to enable comparisons to be made to younger athletes. A mixed method analysis of perceptual fatigue in sight of competition, would provide opportunity to thoroughly consider perceived competition stress, recovery and workload for Masters athletes. This would provide a wider scope of analysis with regards to holistic demands of competition and corresponding perceptual fatigue causes and responses. Such information would support practitioners to develop more specific training approaches which consider the requirements of the sport and the subjective experience (Russell et al. 2024). The aim of this study is to examine the effect of sailing competition on perceived mental fatigue, physical fatigue, stress, recovery and workload, and to explore modulating factors of perceptual fatigue. It is hypothesised that mental fatigue, physical fatigue, stress, and workload will increase while recovery will decrease due to competition compared to the pre‐competition period.

## 2 Materials and Methods

### 2.1 Participants

Twenty‐two Masters athletes (18 male 62.22 ± 8.82 years, 1.79 ± 0.06 m, 77.44 ± 11.19 kg, 4 female 53.75 ± 4.99 years, 1.63 ± 0.05 m, 62.00 ± 3.27 kg) competing in the 2023 Oceania and Australian Laser Masters Championships participated in the study (15 Laser Radial and seven Laser Standard rig‐size sailors). A power analysis was performed for sample size estimation using G*Power 3.1.9.4. Based on a previous study which used Wilcoxon‐signed rank tests to assess pre‐post‐ competition changes in mental fatigue and physical fatigue (Russell et al. 2020), a required sample size of 19 was estimated, for a medium effect size (dz = 0.61) with an alpha of 0.05 and a power of 0.80. An additional three participants were recruited to mitigate for potential attrition (Abt et al. 2020). The rig‐sizes reflect different sail areas which have been introduced in the ILCA to improve accessibility of sailing to a wider pool of athletes. The participants' average number of years competing across all Sailing Classes was 38.36 years (SD = 17.82) and 19.41 years (SD = 13.25) in the ILCA. According to the Participant Classification Framework by McKay et al. (2022), participants would be classified as being in tier two (Trained/Developmental) or tier three (Highly Trained/National Level). All participants provided written informed consent. Ethical approval was granted by the University Human Research Ethics Committee, project ID: HEAG‐H 203_2022.

### 2.2 Procedure

A cross‐sectional time‐series survey design was implemented. Participants completed an online survey during the 48‐h prior to and following competition. The pre‐competition survey included demographic related questions, Visual Analogue Scales (VAS) for perceived mental fatigue and physical fatigue, the Short Recovery Stress Scale (SRSS), the NASA Task Load Index (NASA‐TLX) to measure current and expected workload, questions related to expected timings for various aspects of the competition, and perceived causes (modulating factors) of mental fatigue and physical fatigue in relation to the preparation period. The post‐competition survey included the same questions (VAS, SRSS, NASA‐TLX, actual competition timings, perceived modulators of mental fatigue and physical fatigue) but in relation to experiences during the competition for the VAS and NASA‐TLX, and their current experiences for the SRSS. Each survey was available online via Qualtrics (Qualtrics Provo, UT) and took no longer than 10 min to complete. Participants were emailed the pre‐competition survey 48‐h before the competition commenced, while the post‐competition survey was emailed directly after competition. Participants had 48‐h to complete each survey. On average, the pre‐competition survey was completed 32.58 h (SD = 16.53 h) before competition, with 11 participants completing it 2 days prior to competition, nine participants completing it 1 day prior and two participants completing on the day of the start of the competition. The post‐competition survey was on average completed 14.55 h (SD = 16.00 h) following the last race of competition, with 15 participants completing it on the end day of competition, five participants completing it 1 day after and one participant completing it 2 days after the last race of competition.

### 2.3 Measures

#### 2.3.1 Demographic Information

This section asked questions relating to individual characteristics (name, self‐reported gender, year of birth, individual height and body mass, club representation, and intended competition rig) and training history (previous performance history such as experience, physical and mental preparation, as well as previous results). Questions regarding gender identification were consistent with definitions provided in the World Sailing Transgender and Non‐Binary Eligibility Policy.

#### 2.3.2 Mental and Physical Visual Analogue Scales (VAS)

Visual Analogue Scales were applied in both pre‐ and post‐competition surveys to examine changes in perceived mental fatigue and physical fatigue due to competition. Visual analogue scales have been validated for measuring perceptual fatigue (e.g., Visual Analogue Fatigue Scale [VAFS]; Tseng et al. 2010) and subsequent research in other sports has adapted these to assess perceived mental fatigue and physical fatigue before and after competition (e.g., Russell, Jenkins, et al. 2022). Participants were required to rate their perceived level of mental fatigue and physical fatigue on a slider scale of 0–100 (0 = no fatigue, 100 = maximal fatigue). Each marker on the slider scale initially appeared at the halfway point, and an adjustment was required to count as a result. For the pre‐competition survey participants were asked to rate their current perceived levels of mental fatigue and physical fatigue, and for the post‐competition survey participants were asked to rate their perceived level of mental fatigue and physical fatigue during the regatta.

#### 2.3.3 Short Recovery Stress Scale (SRSS)

The SRSS is an eight‐item subjective monitoring tool assessing recovery‐stress state in a multidimensional manner. It has been previously used in sailing research (Winchcombe et al. 2021) and is a valid and reliable recovery‐stress monitoring tool (Kellmann and Kölling 2019). Recovery domain items included Physical Performance Capability, Mental Performance Capability, Emotional Balance, and Overall Recovery. Stress domain items included Muscular Stress, Lack of Activation, Negative Emotional State and Overall Stress. Using a seven‐point scale ranging from 0 (does not apply at all) to 6 (fully applies), participants rated how they felt in relation to their ‘best recovery’ or ‘highest stress state’. For both the pre‐competition and post‐competition survey, the SRSS was used to assess the athletes' current recovery‐stress state, and not during competition.

#### 2.3.4 NASA Task Load Index (NASA‐TLX)

The NASA‐TLX provides an overall workload score for an activity based on Mental Demands, Physical Demands, Temporal Demands, Own Performance, Effort and Frustration. Using a 21‐gradation scale (i.e., 0–20), participants described workload in relation to ‘very low’, ‘medium’ and ‘very high’ increments. The NASA‐TLX has been used in previous sailing research to measure mental workload (Laera et al. 2023). The pre‐competition survey included questions phrased around their current and expected workload whereas the post‐competition survey included questions related to the actual perceived workload experienced in competition.

#### 2.3.5 Expected and Actual Competition Timings

Participants were required to estimate the anticipated (pre‐competition survey) and actual (post‐competition survey) time spent undertaking various aspects of competition, including Off‐Water Physical, Mental and Psychological Pre‐Race Race Routines, and On‐Water Pre‐Race Routines. Estimated timings were reported in minutes (mins).

#### 2.3.6 Perceived Causes (Modulating Factors) of Mental Fatigue and Physical Fatigue

A list of potential causes of fatigue (training/planning preparation, sleep quality, travel, weather conditions, complexity of decisions) were displayed to participants based on previous research (Russell, Jenkins, Rynne, et al. 2019). Participants were required to rate the level of impact each cause had on their mental fatigue and physical fatigue pre‐ and post‐competition using a five‐point rating scale (1 = no impact, 2 = minimal impact, 3 = moderate impact, 4 = significant impact, 5 = major impact). The selection of a five‐point Likert scale was important to support clearer differentiation of responses, as previously described in the literature (Russell, Jenkins, et al. 2022). The post‐competition survey also included several open‐ended questions for participants to complete related to perceived causes of mental fatigue and physical fatigue, such as whether overall performance was influenced by mental fatigue or physical fatigue, and which strategies could have been implemented to improve physical/mental preparation for the regatta. These explorative questions generated qualitative data which complemented the quantitative data and provided additional insight into the perceived causes of fatigue.

### 2.4 Data Analysis

Data was exported from Qualtrics (Qualtrics Provo, UT) to Microsoft Excel where it was prepared for statistical analysis. This included applying methods of error detection and consistency checks (reviewing raw data for errors and omissions), management of missing responses (reviewing and deleting omissions if deemed inappropriate), assignment of unique identifiers for multiple response variables and calculation of difference scores for pre‐post survey comparisons. Quantitative data was then transferred to SPSS (IBM SPSS Statistics 29) for analysis. Following normality testing, Wilcoxon signed‐rank tests were conducted to compare differences between pre‐ and post‐competition in VAS ratings of perceived mental fatigue and physical fatigue, Short‐Recovery and Stress Scale scores, NASA‐TLX ratings, and expected and actual competition timings, as well as Likert ratings for the impact of modulating factors on fatigue due to competition. Alpha level was set at *p* < 0.05. Responses from the open‐ended questions were used as an additional component to support the quantitative data and assist with the interpretation of findings.

## 3 Results

### 3.1 Perceived Physical and Mental Fatigue

**FIGURE 1:**
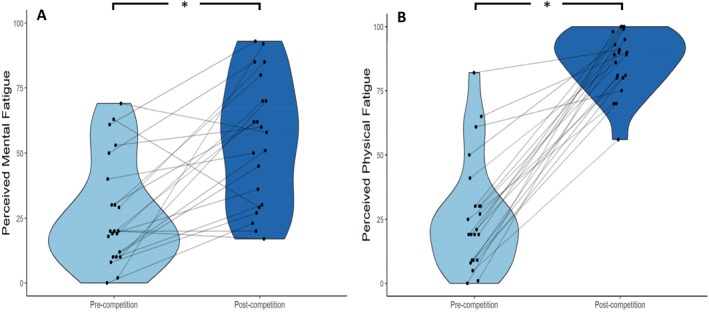
Violin plots displaying participants perceived (A) mental fatigue and (B) physical fatigue pre‐ and post‐competition, measured using a visual analogue scale (0 = no fatigue, 100 = maximal fatigue). Each plot illustrates the distribution of individual participant ratings across both timepoints. *Significant difference pre‐to post‐competition.

Results of the Wilcoxon signed ranks tests revealed that Masters athletes rated perceived mental fatigue, *z* = −3.32, *p* < 0.01, *D* = 2.00, and physical fatigue, *z* = −4.02, *p* < 0.01, *D* = 3.32, as significantly higher in the post‐competition survey compared to the pre‐competition survey (see Figure 1).

### 3.2 Perceived Recovery and Stress

Results of the Wilcoxon signed ranks test for the Short Stress Scales revealed a significant difference between pre‐ and post‐competition for Muscular Stress *z* = −3.30, *p* < 0.01, *D* = 1.99, Lack of Activation, *z* = −3.53, *p* < 0.01, *D* = 2.29, Negative Emotional State, *z* = −2.14, *p* = 0.03, *D* = 1.03, and Overall Stress State, *z* = −3.40, *p* < 0.01, *D* = 2.11, with all measures higher post‐competition (see Table 1).

**TABLE 1:** Perceived stress and recovery scores on the SRSS subscales before and after sailing competition.

	Pre‐competition			Post‐competition		
	Mean	SD	Median	Mean	SD	Median
Short stress scales						
Muscular stress^a^	2.50	1.30	2.50	4.24	1.18	4.00
Lack of activation^a^	1.36	1.59	1.00	3.48	1.36	4.00
Negative emotional state^a^	1.23	1.27	1.00	1.90	1.45	2.00
Overall stress state^a^	1.82	1.50	1.50	3.76	1.09	4.00
Short recovery scales						
Physical performance capability^a^	4.00	1.02	4.00	2.38	1.07	2.00
Mental performance capability^a^	4.18	0.96	4.00	3.38	1.24	4.00
Emotional balance	4.45	1.06	4.00	4.00	1.41	4.00
Overall recovery^a^	4.09	1.06	4.00	2.52	1.17	3.00

^a^ Significant difference between pre‐ and post‐competition.

Results of the Wilcoxon signed ranks test for the Short Recovery Scales revealed a significant difference between pre‐ and post‐competition for Physical Recovery State, *z* = −2.37, *p* = 0.02, *D* = 1.17, Mental Performance Capability, *z* = −2.37, *p* = 0.02, *D* = 1.17, and Overall Recovery State, *z* = −3.23, *p* < 0.01, *D* = 1.89 (see Table 1) with all measures higher pre‐competition. No difference was observed for Emotional Balance, *z* = −1.33, *p* = 0.19, *D* = 0.59.

### 3.3 Perceived and Expected Workload

**FIGURE 2:**
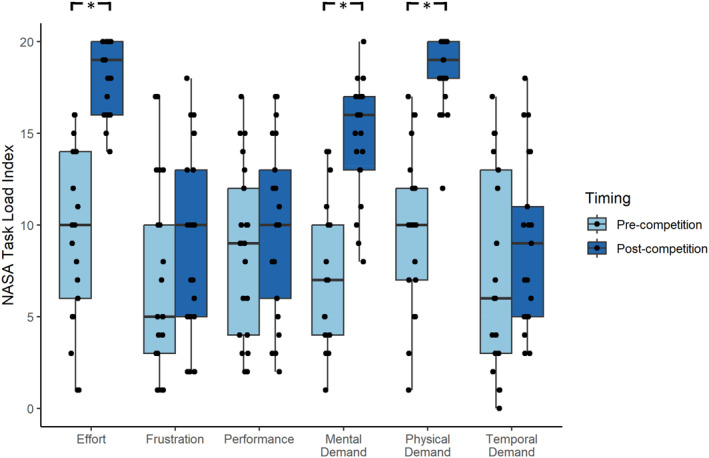
Box plots displaying participants perceived workload pre‐ and post‐competition, measured using NASA TLX (0 = very low, 20 = very high). Each plot illustrates the distribution, median, and interquartile range of ratings across both timepoints. *Significant difference pre‐ to post‐competition.

Results of the Wilcoxon signed ranks tests revealed that NASA‐TLX scores were significantly higher in the post‐competition survey compared to pre‐competition for Mental Demand, *z* = −3.79, *p* < 0.01, *D* = 2.75, Physical Demand, *z* = −4.03, *p* < 0.01, *D* = 3.35, and Effort *z* = −4.02, *p* < 0.01, *D* = 3.34 (see Figure 2). No significant differences were observed for temporal demand, performance and frustration (*p* > 0.05). No significant differences were observed between the participants expected workload of competition, estimated pre‐competition, and the actual perceived workload in competition, estimated post competition (*p* > 0.05; See Table 2).

**TABLE 2:** Expected and actual perceived workload and timings experienced in sailing competition.

	Expected (Pre‐Competition)		Actual (Post‐Competition)	
	Mean	SD	Mean	SD
NASA‐TLX (workload)				
Mental demand	13.64	3.30	14.81	3.17
Physical demand	17.64	1.73	18.33	2.06
Temporal demand	9.23	4.22	9.05	4.49
Performance	8.14	3.60	9.95	4.76
Effort	18.50	1.57	18.10	1.92
Frustration	7.91	4.20	9.00	5.23
Perceived timings (minutes per day)				
Off‐water physical pre‐race routine^a^	18.41	19.72	8.48	9.96
Off‐water mental pre‐race routine	29.55	30.86	25.71	23.57
Off‐water psychological pre‐race routine	11.36	18.53	5.95	8.61
On‐water pre‐race routine	32.27	16.95	29.29	12.97
Total on‐water sailing time	224.77	55.43	203.73	45.19

^a^ Significant difference between expected and actual time spent on task.

### 3.4 Expected and Actual Competition Timings

Results of the Wilcoxon signed ranks test revealed the expected time spent completing Off‐Water Pre‐Race Physical Race Routines was significantly longer than the actual time completed in the regatta, *z* = −2.59, *p* = 0.01, *D* = 1.33. No difference was observed for On‐Water Sailing Time, On‐Water Pre‐Race Routines, Off‐Water Mental Pre‐Race Routines and Off‐Water Psychological Pre‐Race Routines (*p* > 0.05; see Table 2).

### 3.5 Perceived Modulators of Fatigue

**FIGURE 3:**
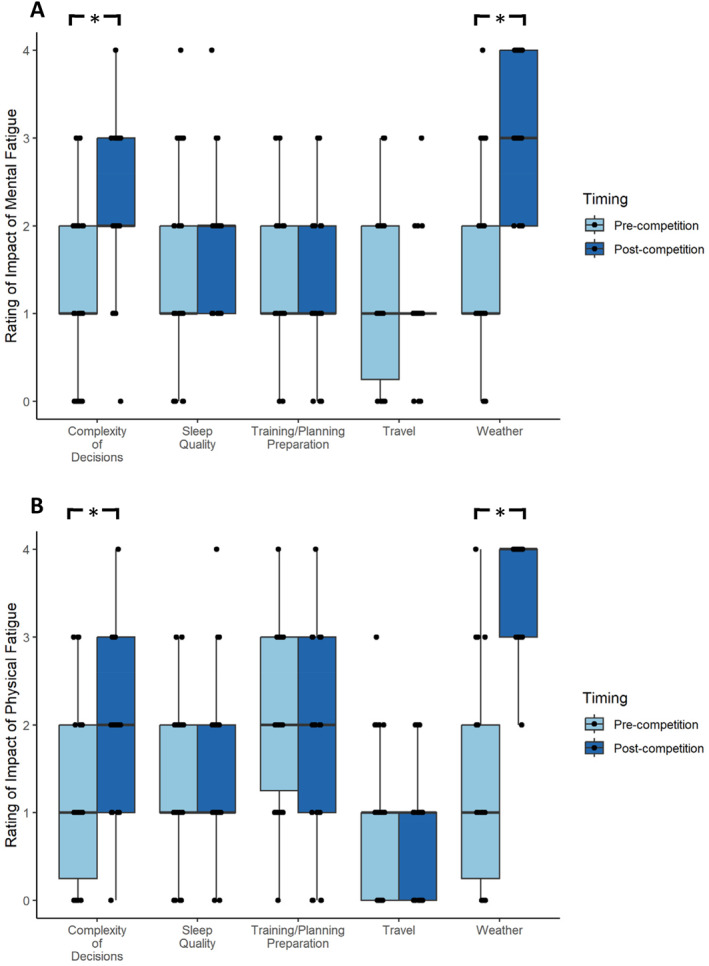
Box plots displaying participants' rating for the impact of modulators on (A) mental fatigue and (B) physical fatigue pre‐ and during‐competition. Level of impact was rated on a five‐point Likert scale (1 = No impact, 2 = 245 Minimal Impact, 3 = Moderate Impact, 4 = Significant Impact, 5 = Major Impact). Each plot illustrates the distribution, median, and interquartile range of ratings across both timepoints. *Significant difference pre‐ to post‐competition.

For mental fatigue, participants rated the impact of Weather Conditions, *z* = −3.58, *p* < 0.01, *D* = 2.36, and Complexity of Decisions, *z* = −2.79, *p* < 0.01, *D* = 1.48, as significantly higher in the post‐competition survey compared to pre‐competition (Figure 3a). There were no significant differences for the other perceived modulators (*p* > 0.05). Similarly, Weather Conditions, *z* = −3.87, *p* < 0.01, *D* = 2.91, and Complexity of Decisions, *z* = −2.34, *p* = 0.02, *D* = 1.15, were rated as having significantly more impact on physical fatigue in the post‐competition survey compared to pre‐competition (Figure 3b). The other modulators showed no significant differences (*p* > 0.05).

Responses to the open‐ended questions support the quantitative data and provide additional insights into the perceived causes of mental fatigue and physical fatigue, as well as strategies to combat these. The tables in the Supplementary Materials present the themes and subthemes generated from the open‐ended questions with example quotes included for each subtheme.

In relation to perceived modulators of mental fatigue and physical fatigue (Supplementary Materials ‐ Table 1), *Environmental Conditions* was a theme with subthemes of wind, wave, and tidal conditions. The *Regatta Format* was also a theme as a modulator of mental fatigue and physical fatigue, with the race day format and the regatta length as the two subthemes within this. Finally, *Physical Conditioning* and *Mental Conditioning* were the final themes for physical fatigue and mental fatigue, respectively.

With regards to strategies that could be implemented prior to competition (Supplementary Materials ‐ Table 2) to improve physical preparation the main theme was *Physical Conditioning* which included general (off‐water) training and sport‐specific (on‐water) training. For mental preparation, *Psychological Skills Training* was the main theme, with subthemes of confidence, motivation, and visualisation. For both physical and mental preparation, *Planning/Strategy* also was a theme.

## 4 Discussion

The purpose of this study was to provide novel insight into perceptual fatigue in sailing. To our knowledge, this is the first study to examine perceived levels of mental fatigue and physical fatigue of Masters sailors before and after an Olympic ILCA competition. In line with the aims of the study, mental fatigue and physical fatigue increased following sailing competition. This change in perceptual fatigue was accompanied by elevated levels of perceived stress and workload, and reduced recovery. Expected workload and competition timings were similar to perceived workload and timings experienced in competition, albeit that participants did expect significantly greater time allocated to off‐water pre‐race physical routines than what was experienced. Complex decision making, perhaps due to the regatta format and/or environmental factors, such as the weather, were identified as important modulators of fatigue in competition. These findings provide insight into the impact of sailing competition on perceptual fatigue states and may be utilised by practitioners to develop well‐rounded training approaches.

Sailing competition induced elevated levels of perceived mental fatigue and physical fatigue, as evident in the VAS measures, which is consistent with research in other sports (Mariano et al. 2023; Russell et al. 2020). Russell et al. (2020) had netballers report perceived mental and physical fatigue using a VAS pre‐ and post‐match on 12 separate competition occasions and showed that while both mental and physical fatigue increased post‐competition, a low proportion of the variance in mental fatigue change could be explained by the variance in physical fatigue, with 86.1% remaining unexplained. The current study supports this finding as the descriptive data indicated that in competition, participants perceived greater levels of physical fatigue compared to mental fatigue. This suggests athletes can distinguish between components of perceptual fatigue (i.e., mental and physical), and experience differing levels of these, during sailing competition. This finding contradicts traditional definitions of fatigue, but supports more contemporary research, such as the State Fatigue Framework (Behrens et al. 2023), where mental fatigue and physical fatigue are described to be largely different constructs albeit with some overlap. Such findings have important theoretical and practical implications, as distinct states of fatigue should ideally be monitored separately by researchers and practitioners, with the aim of developing specific strategies to manage mental fatigue and physical fatigue during competition (Russell et al. 2020).

Within 48 h after sailing competition, athletes reported reduced recovery and increased stress compared to 48 h before competition, which supports previous research in football (Pelka et al. 2018) and netball (Russell et al. 2021). Russell et al. (2021) used the SRSS to observe recovery and stress across a 3‐day, 4‐match netball tournament and found decreased recovery, specifically related to physical performance and emotional balance, as well as increased stress, related to muscular stress and negative emotional state. Within sailing, Winchcombe et al. (2021) is the only one that has analysed perceptual fatigue, recovery and stress experienced in competition. Using the SRSS, the study reported increased perceptual fatigue responses over time in more demanding regatta schedules, and variations in stress and recovery between regattas was attributed to differences in recovery time between racing days. The current study expands the scope of this research to demonstrate perceived recovery may not be complete until multiple days post‐competition.

It is important to note the current study focussed on Masters athletes, who may have different experiences of perceptual fatigue and recovery compared to younger athletes. For example, Fell and Williams (2008) suggests Masters athletes recover more slowly, and experience greater sensations of muscle damage compared with younger counterparts. These age‐related differences are debated though with some studies finding no significant difference in fatigue or recovery between Masters and younger athletes (Schmidt et al. 2021). The level of participation (e.g., elite, sub‐elite, recreational) may be a key moderating factor, as the physical and psychological demands are generally less intense at the lower levels due to shorter event durations and less rigorous training requirements compared to at the elite level where the competition and training demands are significant with regards to both the on‐water and off‐water volume and intensity (Winchcombe et al. 2025). Further research that monitors changes in mental and physical fatigue across multiple time points during and after several competitions with different age groups and participation levels is warranted to determine the recovery and stress time course post sailing competition and whether this is influenced by age or experience. Findings from the current study provide insight into pre‐post competition changes of perceived recovery and stress for Masters athletes, which are important mechanisms for practitioners to consider when planning tailored regenerative strategies between consecutive regattas (e.g., managing training load, implementing cognitive coping techniques) (Kellmann et al. 2018). Practitioners are encouraged to intentionally monitor both mental and physical fatigue during and after competition and to manage this accordingly for each individual athlete to facilitate greater recovery.

Interestingly, there was no significant change in emotional balance before and after the sailing competition. This suggests the competition period did not affect whether athletes felt pleased, stable, in a good mood and in control. The finding that the participants' expectations of competition, workload and timings were akin to those experienced in competition suggests good stability and a high level of control over what occurred in competition. These outcomes support findings from Pelka et al. (2018) who implemented the SRSS with football players and reported that the competition period did not elicit changes in emotional‐related responses. Where a compromise in emotional balance occurred, the authors attributed this to inadequate actioning of coping mechanisms in sight of potential demands deemed as threatening. Maintaining a stable emotional state is important in reducing associated deleterious conditions such as non‐functional overreaching and overtraining (Kellmann and Kölling 2019) and has been identified as a significant aspect in yielding optimum sailing performance (Brandt et al. 2016). The current study highlights sailors were able to maintain a state of emotional balance similar to what was expected during the preparation period, which may be achieved by accurately anticipating the competition timings and subjective workload demands experienced in competition (Kellmann and Kölling 2019; Russell et al. 2021).

In the post‐competition survey, athletes reported increases in subjective ratings of effort, mental demand and physical demand on the NASA‐TLX. These findings align with a study in rugby by Mariano et al. (2023) who reported higher changes in perceived mental fatigue from pre‐to post‐game in backs compared to forwards and this was matched by higher mental and physical demand on the NASA‐TLX among backs than forwards (see also Irvine et al. 2022). This is important as changes in workload related behaviours correspond with exercise tolerance (Behrens et al. 2023) and recovery‐stress balance (Kellmann et al. 2018). Interestingly, subjective feelings of frustration, performance, and temporal demand on the NASA‐TLX remained stable in both preparation and competition. In line with the finding that emotional balance remained stable, it appears the changes in perceptual fatigue reported by the sailors in the current study were not underpinned by changes in emotions, such as frustration. The State Fatigue Framework (Behrens et al. 2023) suggests these are important mechanisms of perceptual fatigue, but the current findings imply that the competition period in sailing did not significantly affect the pace, emotions and sense of accomplishment associated with task demands. From a practical perspective it would be important to monitor these factors across multiple competitions, with varying demands and performance outcomes, as this may be particularly useful in preventing performance stagnation, maladaptation, long‐term under‐recovery and achieving optimal competition performance (Kellmann and Kölling 2019).

As well as examining changes in the underlying mechanisms of perceptual fatigue, the present study investigated the modulators associated with mental fatigue and physical fatigue. The causes of mental fatigue and physical fatigue were described to be largely different constructs in both the quantitative and qualitative responses. It was identified that complex decision making, perhaps due to the regatta format and/or environmental factors, such as the weather, are important modulators of fatigue during competition. This finding aligns with research suggesting unexpected changes in the environment, over‐analysis and information processing induce fatigue in sport settings (Russell, Jenkins, Rynne, et al. 2019). Qualitative responses also provided insight into potential strategies which could be implemented to combat increases in mental fatigue and physical fatigue during competition. Of note were the suggestions for greater physical conditioning through general (off‐water) training and sport‐specific (on‐water) training, and psychological skills training to enhance confidence, motivation, and visualisation leading up to a competition. Future research should explore these potential strategies through the development of robust intervention studies as these could have important implications for practitioners working with athletes (Russell, Jenkins, Smith, et al. 2019; Weiler et al. 2025).

While this novel study has several important findings, there are limitations which must be acknowledged. Most noteworthy, findings are directly related to the sample population, level of competition, regatta duration and weather conditions evident at the time of study, potentially limiting the generalisability of the study to other contexts (e.g., different ages, competitions, sports). Varying responses may occur in regattas where there are different demands, expectations, and pressures. Moreover, despite best efforts to get participants to complete the survey as close to competition as possible, the timing of the survey will have influenced the results given the potential issues related to retrospective recall and the impact of other external factors. Future research could expand on present findings to explore perceived responses across different time periods, levels of experience, age, and regattas where varying environmental conditions and regatta formats exist. This would provide more insight into the patterns of perceived responses and how individual and contextual factors influence this process. To aid with specificity, a list of modulators of fatigue were provided to participants based on previously validated literature (Russell, Jenkins, Rynne, et al. 2019; Russell, Jenkins, Smith, et al. 2019). This may have restricted the scope of analysis to the list of modulators included. Future research could utilise in‐depth interviews directly before and after competition to explore other perceived modulators of fatigue in sailing in a more holistic manner and take in to account how context mediates the impact of these modulating factors (Russell, Jenkins, Rynne, et al. 2019). Finally, the impact of perceptual fatigue on performance is identified as an important dynamic within broader literature (Russell, Jenkins, Smith, et al. 2019; Van Cutsem et al. 2017), which was not examined in the current study; as such, future research could expand current findings to explore this relation.

## 5 Conclusion

This study is the first to present perceived fatigue, stress, recovery, workload and modulators of fatigue experienced by ILCA Masters athletes before and after sailing competition. Findings indicate sailing competition induces distinct elevations in perceived mental fatigue and physical fatigue entities, and this is underpinned by elevated stress, workload and reduced perceived recovery post‐competition. Weather conditions and complex decision‐making were found to be modulating factors of perceived mental fatigue and physical fatigue during sailing competition. These multivariable findings provide a thorough analysis of the holistic demands of competition and corresponding perceptual fatigue causes and responses. Collectively, these insights may be considered by practitioners to support the development of tailored interventions which address fatigue and performance.

## Funding

The authors have nothing to report.

## Conflicts of Interest

The authors declare no conflicts of interest.

## Supporting information


Supporting Information S1
