# Supplementary material for: Perceived Mental and Physical Fatigue, Stress and Recovery, and Workload in Masters Athletes Pre‐ and Post‐Sailing Competition
Source: Eur J Sport Sci. 2025 Dec 20;26(1):e70082. doi: 10.1002/ejsc.70082 (PMC12717789; doi:10.1002/ejsc.70082)
Supplement: Supplementary file 1 — Supporting Information S1 [file EJSC-26-e70082-s001.docx]

# **Supplementary Materials**

Table 1. *Themes and subthemes generated from the qualitative responses to the open-ended questions related to perceived* *modulators of mental and physical fatigue during competition. Example quotes included for each subtheme.*

| ***Question*** | ***Theme*** | ***Subtheme*** | ***Data Extract*** |
| --- | --- | --- | --- |
| *Do you think your overall performance was influenced by physical fatigue across the regatta period. (If yes, please describe why)* | *Environmental Conditions* | *Wind Conditions* | *“Strong winds, beyond what I could physically handle”* |
|  |  | *Wave Conditions* | *“Days 2 and 3 had 3 races in full hiking [with] significant wave conditions”* |
|  | *Physical Conditioning* |  | *“Most races I was at exhaustion by the end or during”* |
|  |  |  | *“Significant impact on ability to hike out and maintain boat performance”* |
|  | *Regatta Format* | *Race Day Format* | *“3 race days, performance was great in 1^st^ race, fell off for next two races”* |
|  |  | *Regatta Length* | *“Less ability to hike and move sheet on the last day”* |
| *Do you think your overall performance was influenced by mental fatigue across the regatta period. (If yes, please describe why)* | *Environmental Conditions* | *Wind Conditions* | *“Very stressful regatta as the racecourse was quite complex with current and shifty conditions at times”* |
|  |  |  | *“Complex wind patterns”* |
|  |  | *Tidal Conditions* | *“Complex tide patterns”* |
|  | *Mental Conditioning* |  | *“Decision making in the last race was not to usual standard. Attribute that to general exhaustion”* |
|  | *Regatta Format* | *Race Day Format* | *“Decision making was worse in later races each day”* |
|  |  | *Regatta Length* | *“After failing to handle conditions on day one, moved to preservation mode instead of performance mode”* |

**Table 2.** *Themes and subthemes generated from the qualitative responses to the open-ended questions related to strategies that could be implemented to improve physical and mental preparation. Example quotes included for each subtheme.*

| ***Question*** | ***Theme*** | ***Subtheme*** | ***Data Extract*** |
| --- | --- | --- | --- |
| *Were there any strategies you could have implemented to improve your physical preparation for the regatta (If applicable, provide details)* | *Physical Conditioning* | *General (off-water) Training* | *“More fitness training, strength and endurance”* |
|  |  |  | *“More exercises for upper body strength”* |
|  |  |  | *“More aerobic training sessions”* |
|  |  | *Sport-Specific (on-water) Training* | *“Longer on water sessions on consecutive days in the couple of weeks leading in. Three races per day was quite demanding”* |
|  |  |  | *“More training in heavy winds”* |
|  |  |  | *“More time in the boat”* |
|  | *Planning/Strategy* |  | *“More study of the wind forecast, terrain map, water current map”* |
| *Were there any strategies you could have implemented to improve your mental preparation for the regatta (If applicable, provide details)* | *Psychological Skills Training* | *Confidence* | ***“****Having not done major regattas for a while I lacked a bit of confidence and self-belief”* |
|  |  | *Motivation* | *“It wasn’t considered an important regatta, so I didn’t do much mental preparation”* |
|  |  |  | *“Not an issue as expectations low”* |
|  |  | *Visualisation* | *“Visualization of starting sequence, better planning of race scenarios”* |
|  | *Planning/Strategy* |  | *“Better planning of race scenarios”* |
